# New Jersey State Dental Society

**Published:** 1891-03

**Authors:** 


### NEW JERSEY STATE DENTAL SOCIETY.—TWENTIETH ANNUAL SESSION.

(Continued from page 125.)

*Professor Charles Mayr.*—I have listened with very much pleasure to the reading of Dr. William Trueman's paper and the remarks of Dr. James Truman upon it, and although I am not a practising dentist, I was once consulted by a well-known practitioner in regard to the case of a lady, about thirty-five years of age, in whose mouth the entire surface of the lower and upper incisors were gone. I could not explain the case satisfactorily. There lingered in my mind a suspicion that it might have been a case of polishing and embellishing the teeth until they were too far destroyed. It was then nine o'clock in the morning, but I used some litmus paper, which showed the presence of a non-volatile action around the lips. The surface was very hard, a circumstance which can be very well explained, and being an intellectual lady, she was no doubt fond of talking, and the polishing of course was more active for that reason. I suggested that it might be good to have a kind of a mat for the teeth,—that is, some kind of a coat fitting over them made of some material which would prevent the action of acid upon them, so that in the mean time the diseased condition might cease. I do not know whether that suggestion was followed, but it appeared to me at that time to be the best solution. Perhaps some of you gentlemen may know how to utilize such a suggestion. Dr. Truman no doubt has the right idea. I merely suggested it as a kind of protective cap in the hope that, in the mean time, the tooth would not lose any more, but might possibly regain a little.

Dr. Trueman used the expression that it would be "reasonable to suppose" that certain things would be. I have very great respect for laboratory investigations, and am very far from depreciating them at all, but in science, in my opinion, there is nothing that is "reasonable to suppose." You cannot suppose anything; you must have your actual facts; and then, if you make a supposition (whether it is reasonable or not is immaterial), follow it out and work on the facts, and see if they harmonize; whether it is reasonable that they should or not is to me indifferent. So I think any tendency to supposing is very detrimental to your progress. Experiments made in the dentist's chair are far more valuable than those made in the laboratory. A dentist can collect a thousand facts before we can get in the laboratory a single case of proof. When decay is produced in a laboratory experiment, I do not consider that true decay; it is only an appearance of decay. Just as you can have an apparently perfectly good two-dollar bill manufactured in Jersey City by some enterprising Italians, but it is not a true note; thus it is with laboratory experiments. I consider anything done in the dentist's chair far superior to anything done in the laboratory. The dentist has to observe, and we can only compare the results, and so I think that Dr. Truman is ahead of the chemists and the biologists in his investigation.

*Professor Peirce.*—I listened to the paper of Dr. William H. Trueman with a great deal of interest, and was glad to follow him in his distinctions as he commenced to read the paper, and was sorry afterwards that he lost that distinction and blended the conditions, which he first described, as the result of one force. We sometimes can learn from the lower animals, and those of you who are familiar with the mouth of a horse know very well that the incisors have deep depressions in the centre, and we judge of the age of the animal as those teeth wear, and at a certain age all the concave space is blotted out by attrition or worn away. That is a very admirable illustration of what I should call abrasion, and it is exactly the thing which takes place in the human mouth during a period of years where, by the mastication of food, the teeth are abraded or worn down. That may or may not be influenced by the secretions of the mouth. It takes place without any acid secretions in a thoroughly neutral or alkaline mouth. Again, we meet with depressions or grooves on the neck of the tooth where the gum has receded and the brush in cleansing comes in contact with the cementum; this is a case of abrasion with or without the acid, simply the result of the friction of a brush on that soft tissue. This is what I call abrasion and not erosion.

*Professor James Truman.*—How do you know that a brush produced the grooves?

*Professor Peirce.*—In this case I know for this reason: I said to the patient, "You use your brush vigorously and you brush around the mouth, for on the right-hand side of the mouth the teeth are worn considerably, and on the other side not so much; there the grooves are very slight indeed." There is always a difference in the condition of such mouths on the right- and left-hand sides, because the patient uses the brush more vigorously with the right hand, although, if the patient is left-handed, we have the deeper grooves on the other side. I draw these conclusions from the fact that the grooves, where they have been produced by the use of the brush, have been found in that condition. And then there may or may not be some acid process. Therefore I call that abrasion.

I have said that we have the incisors worn off by abrasion. Let us follow that a little farther, and we may have a case where that destruction has gone on so far that when the teeth are closed there is a space between the incisors that the handle of a tooth-brush could be placed in. If the papillæ on the tongue are tested in the morning, we will invariably find an acid condition. Now let us take the crescent-shaped spaces that we so frequently see on the incisors. This may extend two-thirds of the way across the surface of the tooth and may be in depth the sixteenth of an inch. These crescent-shaped spaces arise from acid secretions in certain glands in the lips, and this acidity is due to a certain condition of the glands because, where we notice these cavities, we invariably find the patient belongs to a gouty diathesis. I do not say that in all persons belonging to a gouty diathesis such conditions will always be produced. I have examined the mouths of three young ladies who have the spaces described, all of whom belong to a gouty diathesis, and one is so troubled by it that she is oftentimes confined to her room from the effect of this inherited condition. The other two are entirely free from this disease, but their teeth are nevertheless covered with the crescent-shaped spaces, and I have lined the entire surface of some of the teeth with gold, while on others not more than one-third of the labial surface of the tooth is gone. Sometimes I have taken litmus paper and laid it over the tooth and ascertained the locality as nearly as possible, and then have pinched those glands with my small plyers, irritated them and applied carbolic acid and iodine, and have quite modified the effect. I am satisfied that the destruction is due to a certain condition of the glands in that locality, and that, oftentimes, is quite limited.

I fully concur in the statements of Professor Truman regarding the acid effect. It may be asked whether it is a decay. It is a destruction from an acid condition; it is not caries, because the abrasion of the lip has kept it smooth, and we do not have any accumulation of the organic matter there; that has been swept away with the brush or by the friction of the lips, and yet we have all the results of the acid, and we should have absolute caries were it not for the friction of the brush and the lip, which continually removes the organic matter.

One word, however, as to a point made in Dr. William Trueman's paper. He spoke of the friction, which he called attrition, between the bicuspids and molars in the teeth of people advanced in years, and thought that it was the cause of a caries which is observed oftentimes to attack the teeth after a period of fifteen or twenty years of entire absence of decay, and is more pronounced between fifty-five and sixty years of age. We are all familiar with the fact that we may have caries in early life, then a period of little or no destruction of tissue, and then, at the age mentioned, the surface of the tooth is attacked and the destruction goes on rapidly. I do not believe that friction has anything to do with the progress of decay in that locality. I believe it is due entirely to the fact that with the teeth there is a natural wasting of tissue and a waning of the continuity which has formerly existed between the organic and inorganic matter of the tooth, and from the fact of the change of organic structure in advancing years the lime-walls are rapidly dissolved and carried away, and thus we have the loss of tissue manifested.

He also alluded to the peculiar adaptation of the molars and bicuspids to each other, and attributed the depression of the one and the bulging of the other—their fitting so closely together—to attrition. The fact must be remembered that, though the teeth are dense, there is a possibility of displacement or rearrangement of the molecular structures.

*Dr. William H. Trueman.*—I don't know that I have anything further to add excepting to say that the paper was and is intended to be simply suggestive. In regard to the use of prepared chalk, I have been prescribing it for years on young patients, and have done so with a great deal of success. I have no doubt that in young persons it will do a great deal of good. I have also prescribed it for years in cases of erosion, but have not in a single case found it to be of the slightest effect. There was always a question whether the patient faithfully used it as directed to do; but in a number of cases I have no reason to doubt it has been used conscientiously and constantly and continued for years. I have seen no cessation of the erosion during its application, nor have I seen any effect when they have ceased to use it. In my opinion there is very little absolutely fixed on this question as yet, but if erosion is due to a changing condition of the secretions I would hardly expect the prepared chalk to neutralize the acid. The probability is that the destructive agent (if it be an acid, which it may or may not be) is generated in contact with the teeth, and it probably just as readily acts upon the teeth as it would upon the carbonate of lime, and will be neutralized in that way.

Some years ago Dr. Register read a paper on "The Use of the Atomizer in the Mouth for Cleansing the Teeth," which impressed me very favorably. The apparatus he used was very useful. Within a few months my attention has been called to this arrangement (exhibiting apparatus), which is very inexpensive, costing only seventy-five cents. I think a wash might be used very well with this. It is possible that this might produce some systemic disturbance, but with an instrument of this kind a very small amount of fluid can be brought in direct contact with the tissues of the mouth. This is simply suggestive; I have not used it long enough to pronounce any opinion upon it.

On motion, the paper of Dr. Trueman was passed.

Adjourned until Thursday, July 17, at 10 o'clock A.M.

